# The effect of mere-measurement of cognitions on physical activity behavior: a randomized controlled trial among overweight and obese individuals

**DOI:** 10.1186/1479-5868-8-2

**Published:** 2011-01-11

**Authors:** Gaston Godin, Ariane Bélanger-Gravel, Steve Amireault, Marie-Claude Vohl, Louis Pérusse

**Affiliations:** 1 Canada Research Chair on Behavior and Health, Faculty of Nursing, Laval University, Quebec city (Quebec), Canada; 2 Research Group on Behaviors in the Field of Health, Faculty of Nursing, Laval University, Quebec City (Quebec), Canada; 3 Department of Food Science and Nutrition, Faculty of Agriculture and Food, Laval University, Quebec City (Quebec), Canada; 4 Department of Social and Preventive Medicine, Faculty of Medicine, Laval University, Quebec City (Quebec), Canada

## Abstract

**Background:**

The promotion of physical activity among an overweight/obese population is an important challenge for clinical practitioners and researchers. In this regard, completing a questionnaire on cognitions could be a simple and easy strategy to increase levels of physical activity. Thus, the aim of the present study was to test the effect of completing a questionnaire based on the Theory of Planned Behavior (TPB) on the level of physical activity.

**Methods:**

Overall, 452 overweight/obese adults were recruited and randomized to the experimental or control group. At baseline, participants completed a questionnaire on cognitions regarding their participation in leisure-time physical activity (experimental condition) versus a questionnaire on fruit and vegetable consumption (control condition). The questionnaires assessed the TPB variables that are beliefs, attitude, norm, perception of control, intention and a few additional variables from other theories. At three-month follow-up, leisure-time physical activity was self-reported by means of a short questionnaire. An analysis of covariance with baseline physical activity level as covariate was used to verify the effect of the intervention.

**Results:**

At follow-up, 373 participants completed the leisure-time physical activity questionnaire. The statistical analysis showed that physical activity participation was greater among participants in the experimental condition than those in the control condition (*F*(1,370) = 6.85, *p *= .009, *d *= 0.20).

**Conclusions:**

Findings indicate that completing a TPB questionnaire has a significant positive impact on subsequent participation in physical activity. Consequently, asking individuals to complete such a questionnaire is a simple, inexpensive and easy strategy to increase the level of physical activity among overweight/obese adults.

## Background

Obesity contributes to the development of several chronic diseases and leads to important health-care costs [1]. Consequently, the management of obesity represents an important public health issue. Physical activity is a healthy behavior recommended for the prevention and treatment of obesity [2]. However, despite substantial investments in the promotion of physical activity in the past decade, an important proportion of the population is still inactive [3]. Thus, the promotion of regular participation in physical activity remains an important challenge for researchers and clinical practitioners. In a recent systematic review, it was shown that the effects of interventions aimed at modifying this behavior among obese individuals are quite modest [4]. In this review, a number of reasons were suggested to explain this situation and the authors stressed the importance of identifying efficient approaches to change physical activity behavior among obese individuals.

Recent developments in the domain of health psychology highlight the influence of measurement on the subsequent health-related behavior of individuals [5]. Indeed, a few researchers have observed that a rudimentary exercise such as completing a questionnaire on cognitions regarding a given behavior can actually change this behavior. In this respect, French and Sutton [6] have highlighted the importance of studying this effect for the prediction of habitual health behaviors. This phenomenon, known as the "mere-measurement effect" [7] or "self-generated validity effect", has been observed for a variety of health-related behaviors [8-13], including physical activity [14]. For instance, Williams, Block and Fitzsimons [14] observed that asking participants to answer a single intention question about participation in exercise led to a significant increase in exercise frequency (small-to-medium effect size; *d *= 0.26) at two-month follow-up [15]. This pattern of results has also been observed for commitment to health and fitness assessment [10,11] and for self-reported walking [16], also leading to small-to-medium effect sizes on behavior (*d *= 0.20, 0.28 and η^2 ^= 0.07 respectively). However, these four studies were realized among undergraduate university students and were not based on clear theoretical frameworks, with the exception of the study by Spence et al.[16] which was inspired by the Self-efficacy Theory [17].

Although the mere-measurement effect is more likely to be detected for self-reported behaviors [5], this effect has also been observed for other objectively measured health-related behaviors. In a recent study, Godin et al. [9] reported significantly higher proportions of blood donations among blood donors after the completion of a questionnaire based on an extended version of the Theory of Planned Behavior (TPB). The number of blood donations was extracted from the electronic database of the local organization responsible for the blood drive and this effect was still significant at one-year follow-up. In a subsequent study, Sandberg and Conner [8] replicated this finding among a sample of women asked to complete a questionnaire on cervical screening attendance also based on the TPB [18]. Similarly, attendance was extracted from a database rather than being self-reported. Although the mechanisms by which the mere-measurement effect occurs are not fully understood [6,19], it would appear that completing a TPB questionnaire could lead to a significant increase in future behavior. It must be mentioned that the TPB represents one of the most empirically supported theories of social psychology for the prediction of health-related behaviors, including physical activity [20-23] and has been recently identified as one of the most effective theories to inform an internet-based intervention in the context of physical activity [24].

Thus, to our knowledge, the present experimental study is the first to test the effectiveness of the mere measurement effect among overweight and obese individuals. As such, it offers the potential to identify a novel approach to behavior change.

## Methods

### Design and sample

The participants of this study were involved in a larger six-month longitudinal study on genetic "susceptibility" to obesity [25,26]. The sample was drawn from a population of volunteer adults recruited in the Quebec City metropolitan area via local newspapers and radio advertisements between May 2004 and March 2007. Also, e-mails were sent to university students and employees as well as to hospital and government employees. The inclusion criterions for the study were to be aged between 18 and 55 years and to have a BMI ≥ 25 kg/m^2^. A trained research assistant conducted a 15-minute telephone interview with people who responded to the advertisements. They were then alternatively allocated to the experimental or control conditions depending on the order of the telephone interview. Both participants and interviewers were blind to the objective of the study that is testing a mere measurement intervention; the study was presented as a study on motivation. All participants signed the study consent form approved by the Ethics Committee of the local university.

### Measures

Both groups also completed the Minnesota leisure-time questionnaire to assess baseline levels of physical activity (i.e., daily energy expenditure) [27]. This questionnaire was administered by a trained interviewer who provided participants with detailed instructions and a list of clearly defined physical activities. Participants were asked to indicate whether they had performed or not each physical activity over the last year, when they performed these activities, at what frequency per month and for how long. Overall, the Minnesota leisure-time questionnaire contains 63 items related to sports, recreational, yard and household activities. This instrument presents adequate psychometric values; reliability (test-retest correlation coefficients of .92 and .98) and the correlation coefficients for convergent and concurrent validity were between .33 and .63 when compared to other physical activity questionnaires and .47 with peak oxygen consumption [28].

A second measure of physical activity was obtained at three-month follow-up by means of another previously validated self-administered questionnaire [29,30]. Follow-up LTPA was assessed using the following question: "Within the last three months, how often did you participate in one or more physical activities of moderate intensity, totaling at least 30 minutes in the same day during your leisure time?" Responses were reported on a 7-point scale varying between (1) not at all to (7) four or more times a week. The LTPA psychosocial questionnaire assessed the constructs of an extended version of the TPB that included intention (3 items), perceived behavioural control (PBC) (3 items), attitude (6 items), subjective norm (3 items) and respective beliefs (8 and 5 items respectively). Moreover, additional variables from other theories known to contribute to the explanation of health related behaviors (i.e.: anticipated regret (3 items), moral (3 items) and descriptive (2 items) norms, self-efficacy (3 items), facilitating factors (5 items), and positive feelings (3 items)) were also assessed [31]. All 47 items were presented in reference to the studied behaviour defined as follow: "to regularly participate in one or more physical activities during the next three months". The LTPA psychosocial questionnaire was developed in accordance with guidelines provided by Ajzen [32] and Godin and Kok [20]. The majority of the theoretical constructs presented adequate internal consistency (α = 0.73 to 0.90 or Spearman's r = 0.33 to 0.52, *p *< 0.001), except for positive feelings (α = 0.68) and facilitating factors (α = 0.61) which had moderate internal consistencies.

## Results

### Sample Characteristics

**Figure 1:**
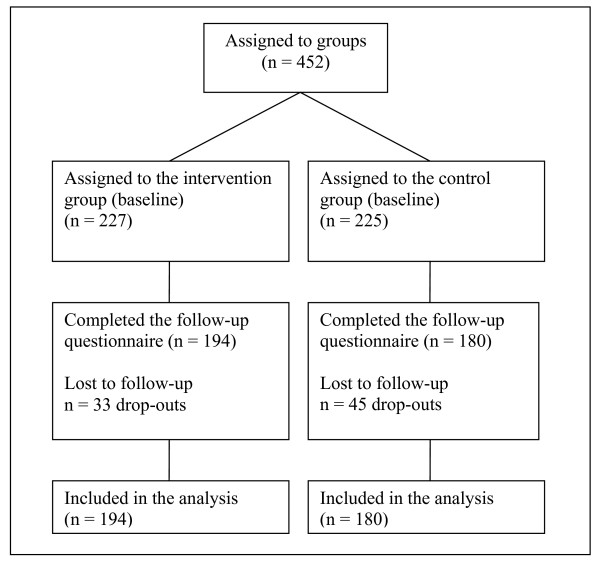
**Flow diagram of participants**.

Of the 452 overweight or obese (all BMI ≥ 25 kg/m^2^) participants who completed the baseline questionnaires, 373 successfully completed the study at the three-month follow-up and were retained for data analysis (Figure 1). The overall attrition rate was 17.5% and did not differ significantly between the two groups. Baseline characteristics of the sample are presented in Table 1. The two groups did not differ significantly in any of the variables assessed at baseline. On average, participants reported to spend 1750-1850 calories per week, suggesting that participants from both conditions were already physically active according to the recent American and Canadian physical activity recommendations (i.e., > 1000-1500 kcal/week) (US guidelines; SCPE). Finally, participants who completed the study did not differ significantly from those who dropped out on all baseline variables, with the exception of gender; women were more likely to drop out than men, *χ*^2 ^(1, N = 452) = 10.6, *p = *0.001. Nonetheless, the results for the mere-measurement effect did not differ when controlled for gender (data not shown).

**Table 1:** Baseline means and standard deviations of the socio-demographic characteristics of the sample by condition (N = 452)

Variables	Control group (F&V)	Experimental group (LTPA)	
	
	*M *(SD)		t-test (*p*-value)
Age (y)	40.3 (10.7)	40.0 (10.7)	0.35 (0.73)
BMI (kg/m^2^)	30.8 (4.6)	31.1 (5.0)	-0.73 (0.47)
Energy expenditure (kcal/d)	263.2 (199.3)	250.4 (211.7)	0.66 (0.51)

	***N *(%)**		**χ**^**2 **^**(*p*-value)**
	
Gender			
Male	110 (48.9)	128 (56.4)	2.55 (0.11)
Female	115 (51.1)	99 (43.6)	
Education level			
Primary/secondary	34 (15.1)	35 (15.4)	0.008 (0.93)
At least collegial	191 (84.9)	192 (84.6)	
Annual income			
< 30,000	90 (40.9)	103 (45.6)	0.99 (0.32)
≥ 30,000	130 (59.1)	123 (54.4)	

### Mere-measurement Effect

An analysis of covariance with baseline level of physical activity as covariate was performed to evaluate the mere-measurement effect of completing a TPB questionnaire. Baseline level of physical activity was included as covariate given that the follow-up measure of physical activity was based on a different instrument. This analysis revealed a main effect for the study condition, *F*(1,370) = 6.85, *p *= .009. Post hoc t-test analysis revealed that the mean score of physical activity level at follow-up in the experimental group (*M *= 5.23, *SD *= 1.61) was significantly higher (*p *= 0.05) than the mean score in the control group (*M *= 4.90, *SD *= 1.63), leading to a significant small effect size (*d *= 0.20, CI_95% _= 0.00-0.41) [15].

## Discussion

Findings of the present study indicate that asking questions about cognitions regarding physical activity positively influence subsequent self-reported participation in this behavior over a three-month period. Moreover, these results compare favorably to a recent meta-analysis that observed short- and mid-term (i.e., six to twelve months of follow-up) small-to-medium effect sizes on self-reported physical activity (SMD = .28, CI_95% _= 0.15 to 0.41) in more intensive interventions among adults [33]. Thus, results from the present study suggest that having only overweight/obese individuals complete a TPB questionnaire that also included other theoretical constructs such as anticipated regret, moral norm, and positive feelings is an easy and inexpensive way to promote exercise, especially in clinical settings where time is scarce (e.g., in the physician's office).

In spite of the growing scientific evidence of the effect of the mere-measurement of cognitions on behavior, the review by French and Sutton [6] highlights the fact that this phenomenon is expected to be small for health-related behaviors and to be mostly observed for non-complex behaviors such as those requiring a single action (e.g., giving blood). However, our results do not support this view given that physical activity is considered to be a complex behavior defined along dimensions of frequency, intensity of practice and duration of exercise sessions. According to French and Sutton [6], this mere-measurement effect is also more likely to be detected when health-related behaviors are subjectively assessed. As such, it is noteworthy that our measure of physical activity was self-reported. However, mere-measurement effects were observed for physical activity assessed either by self-reported or accelerometer (in the corrected model) in a study by van sluijs et al. [5]. There are also few studies that showed this effect for objectively assessed behavior [8,9,34]. According to these latter authors, the question now is not about the existence of mere-measurement, but about what is causing this effect and under which conditions this phenomenon could be observed.

In the present study, the full constructs of the TPB as well as additional variables from other theories were evaluated in the psychosocial questionnaire on LTPA. This questionnaire measured ten theoretical constructs and three sets of beliefs that required about fifteen to twenty minutes to complete. Consequently, individuals were guided towards deliberation about their behavior throughout the questionnaire (i.e., weighting the desirability and feasibility of taking action) that could lead to greater introjections and behavioral resolutions to become more active. Although the design of the present study could not allow any conclusion about the mechanisms underlying this mere-measurement effect, one could surmise that such an effect is less likely to emerge after completing shorter questionnaires. However, previous studies have reported that assessing only intention by means of a few items changes subsequent performance of studied behaviors at follow-up [10,12,35], including physical activity [14]. Others might also infer that this effect might be the consequence of assessing specific cognitions such as those included in the questionnaire of the present study. In this line of thought, Sandberg and Conner [8] observed that measuring anticipated regret (i.e., the anticipation of regret not to perform the behavior) was an important variable responsible for mere-measurement effect on cervical screening attendance. Since anticipated regret was one of the variables assessed in our extended TPB questionnaire, it can be hypothesized that participants in the present study anticipated feelings of regret about not being physically active in response to the questionnaire and, consequently, increased their level of physical activity.

The precise mechanism behind the mere-measurement effect is still a matter of debate [6,19]. At this time, the dominant explanation of this effect is that asking behavioral intention questions heightens the accessibility of the person attitude towards the behavior, which in turns increases the likelihood that the behavior will be performed [36]. Morwitz and Fitzsimons [36] showed that responding to a query about one's purchase intention increases the activation level of one's pre-existing brand attitude. When the respective brand attitude was both highly accessible and positively valenced then participants were likely to choose that brand. On the other hand, when the activated attitude was negatively valenced this led to a decrease in the choice of this brand. However, the mere-measurement effect may only operate among those whose thoughts and feelings are favorably disposed towards the behavior. In a laboratory setting Morwitz and Fitzsimons [36] showed that positive attitudes increased brand choice, while negative attitudes decreased brand choice. Obviously, future research in this direction is needed to elucidate the potential mechanisms of action of the mere-measurement effect on changes in physical activity behavior.

The relatively modest costs and simplicity of the intervention may add to the appeal of mere measurement as an important additional strategy for improving public health via health behavior change. Nevertheless the use of the mere-measurement effect as a mean to promote public health is likely to be limited by the compliance of individuals to complete adequately the questionnaire. Indeed, questionnaire completion appears to be a prerequisite for a mere-measurement effect to occur [8,9,34]. Consequently, an important implication for using the mere-measurement effect to promote health behaviors is that studies will need to maximize completion rates of the questionnaire in order to produce the greatest impacts on the targeted behavior. Further research could also examine the added value of combining the mere measurement effect with interventions that promote questionnaire completion and/or positive attitudes toward the target behavior. For example, Dillman [37] provides guidance on the content and type of cover letter, questionnaire format, token incentive and return envelop that could be used to improve response rates to mailed questionnaire.

Notwithstanding this positive and significant effect of mere-measurement of cognitions, it must be acknowledged that the effect remains small and might benefit from being used in combination with other techniques for behavior change (see Abraham and Michie [38] for a description of a set of theoretical techniques). Moreover, from a public health perspective, if asking questions regarding a given behavior reinforces or induces its adoption, caution should be exercised before asking questions about sedentary behavior. Indeed, Williams, Block and Fitzsimons [14] observed an increase in illegal drug use following the completion of one item about intention to use illegal drugs. Thus, researchers investigating cognitions regarding sedentary behaviors among overweight or obese individuals instead of those related to physical activity participation must be aware that they may cause more harm than good in asking such questions.

Some limitations of this study must be acknowledged. Firstly, the present study was completed among a group of volunteers representing individuals interested in the topic of the study. Thus, this effect might be more important in this kind of sample, given that the cognition scores were relatively high (data not shown). Sprott, Spangenberg and Fischer [11] also investigated the effect of mere-measurement (described as self-prophecy) when the behavior under investigation was considered by the participant as right or wrong. Their results indicated that this effect was more likely to occur among participants with stronger normative beliefs regarding low-fat snack consumption and health and fitness assessment. Secondly, although we used an experimental design, it was not possible to apply an intention-to-treat approach given that different measures of physical activity were used at baseline and follow-up. Thirdly, subjective measures of physical activity were used in the present study, although both tools have been validated. Also, given that in the present study the measure of physical activity at follow-up differed from the measure used at baseline, it is less likely that the size of the observed effect might be attributed to measurement habituation. Nonetheless, it will remain important in future studies to conduct randomized controlled trials with objective measures of physical activity. Finally, additional research should be conducted among other segments of the overweight/obese population, especially among sub-groups with low socio-economic status.

## Conclusions

To conclude, the present study is the first to report the effect of measurement of cognitions on physical activity among a sample of overweight and obese individuals. This adds to the growing evidence that asking questions influences subsequent physical activity behavior. It also paves the way to new approaches for changing behavior, since this method requires low investments in terms of time and money and could easily be integrated into more comprehensive prevention and health promotion programs, and health care practice.

## Competing interests

The authors declare that they have no competing interests.

## Authors' contributions

GG, MCV and LP designed the study and participated in its coordination. SA participated in data collection and performed data analysis, along with ABG and GG. ABG and GG wrote the draft version of the manuscript. SA, MCV and LP critically revised the manuscript. Finally, all the authors read and approved the final version of the manuscript.
